# Pixel-Domain Just Noticeable Difference Modeling with Heterogeneous Color Features

**DOI:** 10.3390/s23041788

**Published:** 2023-02-05

**Authors:** Tingyu Hu, Haibing Yin, Hongkui Wang, Ning Sheng, Yafen Xing

**Affiliations:** School of Communication Engineering, Hangzhou Dianzi University, No. 2 Street, Xiasha, Hangzhou 310018, China

**Keywords:** user-generated images, color feature parameters, color interaction, color uncertainty and saliency, just noticeable difference

## Abstract

With the rapidly emerging user-generated images, perception compression for color image is an inevitable mission. Whilst in existing just noticeable difference (JND) models, color-oriented features are not fully taken into account for coinciding with HVS perception characteristics, such as sensitivity, attention, and masking. To fully imitate the color perception process, we extract color-related feature parameters as local features, including color edge intensity and color complexity, as well as region-wise features, including color area proportion, color distribution position and color distribution dispersion, and inherent feature irrelevant to color content called color perception difference. Then, the potential interaction among them is analyzed and modeled as color contrast intensity. To utilize them, color uncertainty and color saliency are envisaged to emanate from feature integration in the information communication framework. Finally, color and uncertainty saliency models are applied to improve the conventional JND model, taking the masking and attention effect into consideration. Subjective and objective experiments validate the effectiveness of the proposed model, delivering superior noise concealment capacity compared with start-of-the-art works.

## 1. Introduction

With the rapidly emerging user-generated images, it occurs a massive demand for transmission to meet social needs; moreover, user-generated images end up with being received by the human eye. Therefore, it is necessary to study the minimum visual threshold of color images to remove the perceptual redundancy to the greatest extent.

Human visual perception derives from the visual information received by the human eye. Work related to building the perception model inevitably involves exploring visual mechanisms in depth and investigating what features affect visual perception. For instance, Chang et al. [1] utilized sparse feature, which can be seen as the response of neurons in visual cortex that is closely associated with visual perception, to design a perceptual quality metric. Men et al. [2] extracted temporal quality-related features, which addresses the problems caused by temporal variations, to build a feature-combination video quality assessment method. Liu et al. [3] concerned the low-level human vision characteristics and the high-level brain activities that can capture the quality degradations effectively. As an aggregator, Korhonen [4] extracted sets of features, covering a wide variety of different statistical characteristics in both temporal and spatial dimensions, which are capable to model and train several different specific distortions. Moreover, color vision is an important part of the human visual system (HVS) [5,6,7]. After refining the statistical regularities of chromatic perception, Chang et al. [8] proposed independent feature similarity (IFS) that can predict the perceived distortion of color information within a given image. Considering that the information of image structure cannot reflect the color changes between the reference and distorted images, color similarity is also involved in modeling besides extracting gradient information and saliency information [9]. It can be seen that, based on visual perception characteristics, the primary task of constructing a subjective task model is to fully extract the features within visual information that affect perception.

In practical applications, visual perception redundancy exists not only in the luminance component, but also in the chromaticity component [10,11]. Human color perception needs to be integrated into image and video encoding to maintain the quality of color perception while saving more bitrates [12]. Traditional Just Noticeable Difference (JND) models typically consider the luminance adaptive effect and luminance component contrast masking effect of HVS, as well as edge masking and texture masking [13], uncertainty masking [14], pattern masking [15], structural masking [16], and the effect of eccentricity on visual sensitivity [17]. The JND model in the transformation domain also considers the contrast sensitivity function (CSF), which reflects the bandpass characteristics of the human visual system in the spatial frequency domain [18]. Through the full study of HVS, it has been found that the human eye can only focus on a limited area [19], and HVS scans the entire scene and guides the rapid process of the eye to focus on the area with the most information, known as visual attention [20]. The computing resources of the human brain are allocated to high-attention regions rather than low-attention areas, and visual saliency regulates visual sensitivity in different regions [21]. Thus, visual saliency is used to modulate the masking effect in the JND model. For example, after calculating the visual attention map of an image/video, the pixel corresponding to the highest attention level is selected as the foveal region/fixation point of HVS, the other regions are treated as non-foveal areas, and different weighted values are assigned to different regions. Finally, the weighted map is used to modulate the JND profile and calculate the masking value [22]. In the transformation domain, a combined modulation function by considering the aftereffect of visual attention and contrast masking is designed to modulate the CSF threshold of each DCT coefficient with luminance adaptation factor [23]. From the above analysis, it can be seen that in the JND model, the masking effect of the luminance component has been fully studied, whereas the visual saliency model is used to adjust the masking effect.

As we all know, color plays an extremely important role in the way we understand the world. Since the luminance component can be thought of as an achromatic component channel, its properties are correlated with the intensity of the color stimulus [24]. Therefore, color JND model can apply the visual perception characteristics for parameters in the luminance component to the chrominance component. In the color image JND study, Chen et al. [25] obtained the color CSF in the DCT domain and applied it to the spatial–temporal domain JND model. There also exists works to directly calculate the masking effect of luminance on chromaticity components. For instance, [26] proposed a spatial JND model, which first models the masking effect integrating different masking effects. However, it is more suitable for handling luminance components for considering luminance features. In order to obtain a more accurate color JND model, Xue et al. [27] proposed the chromatic JND model (CJND) according to the finding that human color perception is closely related to the density of cones in the retina [28]. Subsequent studies have found that there is a certain masking effect between adjacent areas in color images [29]. Wan [30] used color complexity to calculate the spatial masking effect of color images. In the latest color JND study, Jin et al. [31] considered the characteristics of full-RGB channel, added pattern complexity and visual saliency, and generated a color image JND threshold called RGB-JND.

In summary, although many studies on color JND have been explored, they are mainly based on the three-channel decomposition of color space, or simply regarding chromaticity as a single quantity from the receiving end of the human eye, or directly applying the luminance-related masking effect to the color component. Existing JND models do not consider color features as deeply as luminance components. Driven by these drawbacks, the chrominance component needs to be analyzed as carefully as the luminance component in JND modeling. The above analysis proposes three key questions for spatial color JND modeling. First of all, which color features are extracted? Second, how do we analyze the interaction between color excitations? Third, how do we fuse and quantify the impact of these heterogeneous color features in the perception sense?

In order to tackle these three problems, the color features are elaborated, which are color edge intensity, color complexity, color area proportion, color distribution position, color distribution dispersion, and color perception difference. Then, the interaction between color regions is analyzed from the perspective of visual energy competition to obtain the color contrast intensity. According to the characteristics of HVS perception, these color features can be divided into visual excitation sources and suppression sources, which express masking effects and visual saliency effects, respectively. In this paper, the degree of color uncertainty caused by color complexity and color distribution dispersion is measured by means of information theory and modeled as a color masking model. Visual saliency caused by color contrast intensity is measured, which is modeled as the adjustment weight. In order to be more consistent with HVS characteristics, color saliency is applied to adjust color uncertainty masking so as to participate in the color image JND model. The main contributions of this paper are as follows.

(1) We carefully extract the color features that affect perception in the image, and on this basis, analyze the interaction relationship between color regions from the perspective of visual energy competition; then, accordingly propose color contrast intensity.
(2) According to the characteristics of visual perception, color complexity and color distribution dispersion are regarded as visual suppression sources, and color contrast intensity is regarded as a visual stimulus source. Then, they are unified to information communication framework to quantify the degree of influence on perception.
(3) The color uncertainty and the color saliency are applied to improve the conventional JND model, taking the masking and attention effect into consideration, wherein color saliency serves as an adjusting factor to modulate the masking effect based on color uncertainty.

The rest of the article is organized as follows. The color feature parameters are analyzed in Section 2. In Section 3, the details in color perception modeling and the framework of the proposed JND model are elaborated. The performance of the proposed JND model is demonstrated in Section 4. Finally, the conclusion is drawn in Section 5.

## 2. Analysis of Color Feature Parameters

Abundant studies on color have been involved in the fields of image quality assessment [32,33], salient object detection [34], and visual attention mechanisms [35]. Whereas, many of these color feature parameters have not yet been applied to the JND model. This section first analyzes the color perception feature parameters involved in the existing studies. In order to fuse these heterogeneous color feature parameters and achieve a unified scale metric, this section analyzes the feasibility of using information theory to fuse heterogeneous color feature parameters. In addition, the interaction that exists between color parameters is analyzed from the perspective of visual energy competition, then represented as color contrast intensity accordingly.

### 2.1. Existing Color Feature Parameters

Both color complexity and color edge intensity are local features, which can be obtained directly in pixel units. Regional color features are based on color regions that are generated from homogeneous regions through color clustering.

Color complexity $m_{c}$ is used to describe the intensity of color change in the area around a pixel in the CIELab color space and is calculated as [36]

(1) $$m_{c}=\sum_{i=1}^{8}\left(L_{i}^{2}+a_{i}^{2}+b_{i}^{2}\right)$$

where $L_{i}^{}$, $a_{i}^{}$, and $b_{i}^{}$ denote the results of the convolution by the Lab three-channel components going through the *i* th direction of the gradient operator, respectively.

Color edge intensity $e_{c}$ is used to describe the distinctness of color edges perceived by the human eye and is calculated as [34]

(2) $$e_{c}\left(x,y\right)=\max\left(D_{c^{{}^{\prime }}}\left(x,y\right)\left|c^{{}^{\prime }}\in \left\{rg,gr,by,yb\right\}\right.\right)$$

where $c^{{}^{\prime }}$ represents the four color opponent channels. $D_{c^{{}^{\prime }}}$ is derived from the maximum boundary response in each direction at each position.

To extract regional color features, Gaussian Mixture Model (GMM) is used to extract 12 color components with relatively high percentages, and each homogeneous color component *c* is expressed as a weighted combination of several similar GMM components [37]. A homogeneous color region is obtained by clustering spatially connected homogeneous color pixels, and the weight of homogeneous region position is defined as follows [38]:

(3) $$l_{c}=\exp\left(-9d_{c}^{2}\right)$$

where $d_{c}$ is the average distance between pixels in region *c* and the center of the image, with pixel coordinates normalized to [0, 1].

The color perception difference $\chi_{c}$ is calculated as [39]

(4) $$\chi_{c}=1-\exp\left(-\frac{\Delta \mu }{\vartheta }\right)$$

$\Delta \mu =∥\mu_{c_{i}}-\mu_{c_{j}}∥$ denotes the Euclidean color distance in CIELab, and $\mu_{c}$ is the mean value of color pixels in the homogeneous color component *c*. ϑ is the normalization parameter.

The homogeneous color distribution dispersion $v_{c}$ is measured as follows [37]:

(5) $$v_{c}=\frac{1}{{\left|X\right|}_{c}}\sum_{x}p\left(c|I_{x}\right)\times \left({\left|x_{h}-M_{h}\left(c\right)\right|}^{2}+{\left|x_{v}-M_{v}\left(c\right)\right|}^{2}\right)$$

where ${\left|X\right|}_{c}=\sum_{x}p\left(c\left|I_{x}\right.\right)$, $x_{h}$, and $x_{v}$ are the horizontal and vertical coordinates of the pixel; $p\left(c|I_{x}\right)$ are the probabilities that the Gaussian mixture clusters pixels $I_{x}$ belong to the homogeneous color components *c*; and $M_{h}\left(c\right)$, $M_{v}\left(c\right)$ are the mean horizontal and vertical coordinates of the homogeneous color component regions.

The homogeneous color weight $\rho_{c}$ is defined as the area of that color component in the whole image as follows:

(6) $$\rho_{c}=\frac{num(c)}{m\times n}$$

where *m* and *n* are the length and width of the image, and num(c) indicates the number of pixels occupied by homogeneous colors *c*.

**Figure 1:**
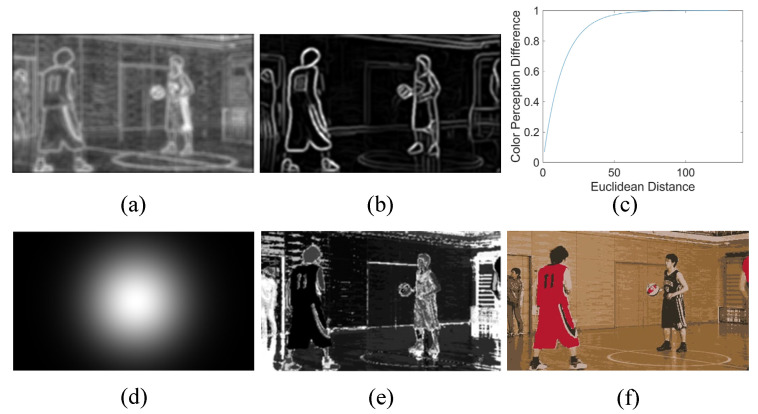
(**a**–**f**) The color complexity $m_{c}$, color edge intensity $e_{c}$, relationship between euclidean distance and color perception difference, saliency weight of color region location $l_{c}$, degree of color distribution dispersion $v_{c}$, and areas of homogeneous color regions, respectively. (Brighter area indicates greater value.)

Figure 1 shows a schematic diagram of each color feature parameter. Table 1 lists these parameters and the corresponding perceptual effects.

### 2.2. Feasibility Analysis of Heterogeneous Color Feature Fusion

The abovementioned color parameters result in the distortion of human eye perception to a certain extent, performing as essential interference factors that affect the accurate perception of the visual system. On the one hand, some excitation sources affect the fixation point of human eyes, thus causing saliency effect, while on the other hand, some feature parameters reduce visual perceptual sensitivity and consume human eyes’ perceptual energy, inducing a masking effect. In order to quantify the extent of the masking and saliency effects, we face the challenge of fusing heterogeneous feature parameters. It naturally leads to the question, can these heterogeneous feature parameters be mapped to the same scale?

In existing studies, refs. [40,41] modeled visual perception as an information communication process in which the visual signal passes through an error-prone communication channel (HVS). The noise level in this communication channel is not fixed, i.e., the HVS does not perceive all information content with the same degree of certainty; then, the amount of information that can be received (perceived) at the receiving end will depend heavily on the noise in the distortion channel (HVS). Therefore, this degree of perceptual uncertainty can be quantified by information theory if a statistical model of the information content can be found [42]. This ideological approach has also been proven to be effective in still image quality assessment (IQA) [43]. Inspired by this, the distortion of human eye perception caused by color feature parameters can be regarded as visual channel noise. If a statistical model of the parameter can be developed based on visual perceptual properties, it can be quantified in view of information theory. Thus, it is feasible to fuse heterogeneous color feature parameters in the information communication framework.

Next, the key point is how to measure equivalent noise in this communication channel. According to [44], information content can be measured by the prior probability distribution and the likelihood function. It has been demonstrated that these measurements are consistent across human subjects and can be modeled using simple parametric functions [42,45]. Motivated by this, this paper adopts the probability distribution function and fitting curve to measure color information content induced by color feature parameters.

### 2.3. Interaction Analysis between Color Feature Quantities

The perceptual effects induced by positive stimuli are known to be positive perceptual effects [46]. Moreover, the transmission and expression of visual information requires energy consumption [47]. Therefore, with limited resource capacity, not all positive stimuli induce positive perceptual effects, i.e., there exists biased competition [48]. The information-theoretic approach can map feature parameters to the same scale for fusion, but it also ignores the problem that the interaction relationship between color feature parameters cannot be expressed. Therefore, in order to obtain a color JND model that is more consistent with HVS, the interactions between color stimuli also need to be analyzed [49].

After the GMM color clustering processing, the image is divided into homogeneous color regions. For color regions, the following visual properties can be observed directly.

(1) With the same dispersion, the larger the homogeneous color area is, the more visual energy allocated to this color area compared with other color areas.
(2) On the condition of same area proportion, if the distribution of one homogeneous color region is more concentrated than that of other regions, it will pose a positive stimulation effect on vision and vice versa.
(3) As the distance between different color regions and the fixation point increases, the competitive relationship gradually weakens.

On the basis of the aforementioned points, the interaction behind these visual properties is defined as color contrast intensity $r_{c}$ in our work.

(7) $$r_{c}=\eta \left(\rho_{c},\Delta v_{c}\right)\cdot l_{c}$$

where $\eta \left(\rho_{c},\Delta v_{c}\right)$ is the intensity of color area competition, which characterizes the visual energy competition caused by the distribution of homogeneous color areas. $\Delta v_{c}=v_{c_{i}}-v_{c_{j}}$ is the difference between the variance of the current perceptually homogeneous color component $c_{i}$ and all others $c_{j}$; so, the larger $\Delta v_{c}$ is, the more dispersed $c_{i}$ is relative to all other color components $c_{i}$, i.e., the less perceptually significant $c_{i}$ is, the weaker the contrast intensity of the current perceptually homogeneous color component tends to be, and vice versa. Based on this perceptual phenomenon, this paper employs $\eta \left(\rho_{c},\Delta v_{c}\right)$, which formulates as

(8) $$\eta \left(\rho_{c},\Delta v_{c}\right)=\left\{\begin{array}{c} \sum_{c_{i}\neq c_{j}}exp\left(\frac{-\Delta v_{c}}{2\rho_{c}^{2}}\right),\Delta v_{c}\geq 0\\ \sum_{c_{i}\neq c_{j}}\left(2-exp\left(\frac{\Delta v_{c}}{2\rho_{c}^{2}}\right)\right),\Delta v_{c}<0 \end{array}\right.$$

Moreover, the intensity of color region competition is also influenced by the proportion of homogeneous color components in the whole image. With the same dispersion, the larger $\rho_{c}$ is, the less the influence of other homogeneous color components on the current component, and the less significant the effect. This is the nut of this model to understand. At the same time, the more η tends to 1 in this equation, the weaker the effect on the color contrast intensity $r_{c}$. Therefore, the model is consistent with visual perception.

## 3. The Proposed JND Model

In this section, color uncertainty and color saliency, of which the proposed JND model is comprised, will be described in detail, and methods to model them will be also presented. Then, the color saliency model is constructed as the modulation factor for the masking effect based on color uncertainty to incorporate in color JND estimation.

### 3.1. Color Uncertainty Measurement

Based on the analysis in Section 2, to measure the degree of color uncertainty in the same scale, parameters can be modeled in the information communication framework. It is known that the essence of color complexity is the degree of local dispersion of color in the pixel domain, which coincides with the concept of entropy in information theory. Moreover, by visually inspecting the reference and distorted images, we observe that the perceptional noise is distributed unevenly over space. For example, compared with the pure-color background, some color sharp-change areas in the images are perceptually more noisy. That is to say, color complexity prevents the visual system from acquiring accurate information, which can be equivalent to the perceptual noise in the visual channel of HVS, as in [45].

Specifically, stimulus intensity is one of the fundamental dimensions of the sensory experience. Understanding the relationship between the physical intensity of a stimulus and the subjective intensity of its associated percept was the main driving force behind the development of the field of psychophysics. This effort was propelled by the finding that the discriminability between two nearby stimuli along a sensory continuum depends only on the ratio between their intensities, not on their absolute magnitudes. This observation was first made by Weber in 1834 [50]. Driven by this concept, relative color complexity $\frac{m_{c}}{\max\left(m_{c}\right)}$ is selected to denote stimulus intensity of $m_{c}$ for perception.

Whence it makes sense to use information entropy to measure the level of perceptual noise caused by color complexity, and equivalent noise of color complexity can be developed as

(9) $$entropy\left(m_{c}\right)=-\sum_{j}p\left(m_{c}\right)\log_{2}\left(p\left(m_{c}\right)\right)$$

(10) $$p\left(m_{c}\right)=1-\gamma_{1}{\left(\frac{m_{c}}{\max\left(m_{c}\right)}\right)}^{\gamma_{2}}$$

where $\gamma_{1}={\left(\max\left(m_{c}\right)-\min\left(m_{c}\right)\right)}^{1-\gamma_{2}}$, $\gamma_{2}$ is the weight assignment factor [36]. As $m_{c}$ tends to $\max\left(m_{c}\right)$, i.e., relative color complexity $\frac{m_{c}}{\max\left(m_{c}\right)}$ tends to 1, the larger the equivalent perceptual noise $entropy\left(m_{c}\right)$ is. Conversely, relative color complexity tends to 0 the smaller $entropy\left(m_{c}\right)$ is. It can be seen that this equivalent model conforms to perception.

**Figure 2:**
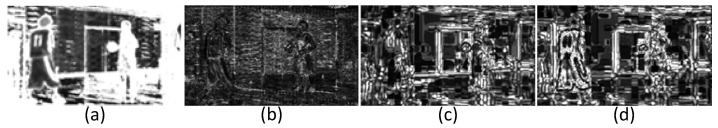
(**a**) Color uncertainty; (**b**–**d**) the color uncertainty masking evaluation in YCbCr three-channels—more details in 3.3 (brighter areas indicate higher uncertainty and masking).

The dispersion of color distribution $v_{c}$ emanates from color variance. The larger the variance, the more dispersive the color distribution is and the more detail the color has. Therefore, the greater the dispersion of color distribution, the more visual energy is consumed, which is equivalent to adding more perceptual noise.

(11) $$p\left(v_{c}\right)=\frac{1}{\sqrt{2\pi }\alpha_{1}}\exp\left(-\frac{{\left(v_{c}-\alpha_{2}\right)}^{2}}{2{\alpha_{1}}^{2}}\right)$$

where $\alpha_{1}$ and $\alpha_{2}$ are both fitting parameters. By using the Nonlinear-additivity model for masking (NAMM) [51] that can eliminate the joint effect of parameters, the color uncertainty *U* is calculated as

(12) $$U=entropy\left(m_{c}\right)-\log_{2}\left(p\left(v_{c}\right)+\epsilon \right)-0.3\cdot \min\left(entropy\left(m_{c}\right),-\log_{2}\left(p\left(v_{c}\right)+\epsilon \right)\right)$$

where ϵ is a very small normal value to avoid the extreme case. Figure 2 shows the color uncertainty and masking effect based on it; the brighter regions indicate a higher degree of uncertainty.

### 3.2. Color Saliency Measurement

Criterion for the existence of a salient object: a salient object is always different from its surroundings, and most likely close to the center of the image [52]. In addition, due to the limited visual energy of the human eye, inevitably there is a competitive relationship between visual excitation sources [48]. Therefore, to research color saliency, it is necessary to consider the degree of color difference and spatial distribution characteristics, even the interaction relationship between color regions. In view of the color itself, the color with higher difference to others attracts more attention. From homogeneous color areas in image, the greater the color contrast intensity, the easier it is to attract human attention. Thereupon, the degree of color saliency can be modeled as follows:

(13) $$\Lambda =\chi_{c}\cdot r_{c}$$

Similar with color uncertainty, the degree of color saliency is quantified by information theory. In particular, areas with higher significance are more noticeable to the human eye and more difficult to conceal distortion. Based on the visual characteristics of the human eye, the probability density function of color saliency $f\left(\Lambda \right)$ is modeled as

(14) $$f\left(\Lambda \right)=\beta_{1}-\frac{\beta_{1}}{1+\exp\left(-\beta_{2}\cdot \Lambda \right)}$$

where $\beta_{1}$ and $\beta_{2}$ are controlling parameters. Experiments show that the color saliency model can distinguish the color region well; however, the color at the edge is affected by the significance of the homogeneous color. If the color saliency of the region is low, the color edge will not be emphasized. Given that the human eye is very sensitive to edges [13], further consideration needs to be given to protect edges. From this, color saliency *I* is calculated as

(15) $$I=-\log_{2}\left(f\left(\Lambda \right)+\epsilon \right)\cdot \Phi \left(e^{c}\right)$$

where ϵ is a tiny positive normal value, $\Phi \left(\cdot \right)$ serves as a filter function, only data greater than a certain threshold are retained, and the rest are taken as 1. Here, edge significance is only considered when the color edge intensity reaches a certain level, and the threshold is determined by subjective experiments.

**Figure 3:**
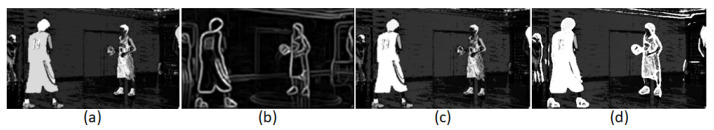
Panel (**a**) denotes Λ, (**b**) is the color edge intensity, and (**c**) is the color saliency without considering the color edge protection. Panel (**d**) accounts for the color saliency of color edge protection (brighter indicates larger value).

For an intuitive effect, the brighter area in Figure 3 indicates greater intensity and greater saliency. These results are in accordance with our perception.

### 3.3. The Proposed JND Model

**Figure 4:**
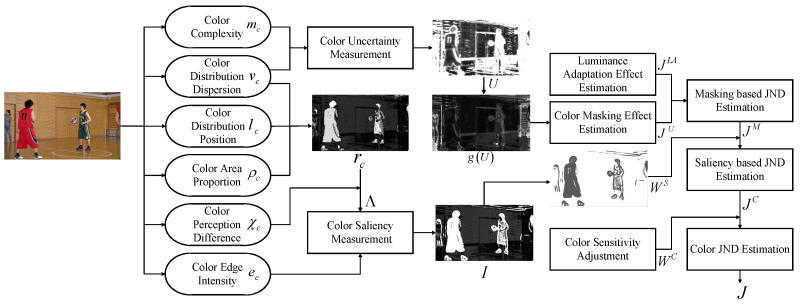
This is the framework of the proposed JND model.

In order to obtain a more accurate JND estimation in color images, the uncertainty model and saliency model need to be considered in our model. Specifically, the color uncertainty performs as a masking effect, and with greater color uncertainty, the more noise can be accommodated, so the masking effect is stronger. Next, the color saliency is envisaged to adjust the masking effect. The stronger the prominence, the more susceptible to human eye attention, so the masking effect of the area is weakened; on the contrary, the masking effect is strengthened. Finally, considering the sensitivity of the human eye to different colors, corresponding perceptual weights are assigned in the three channels of the YCbCr color space. Consequently, the color features and the perception characteristics of the human eye on color have been carefully considered, and a novel JND threshold estimation model for color images is established. The framework for the proposed model is shown in Figure 4, where the brighter area indicates a larger value.

Firstly, the total masking estimation $J_{\theta }^{M}$ is established by considering the luminance adaptive threshold $J^{LA}$ [51] and color uncertainty masking estimation $J^{U}$.

(16) $$J_{\theta }^{M}\left(i,j\right)=J^{LA}\left(i,j\right)+J_{\theta }^{U}\left(i,j\right)-C_{\theta }\times \min\left\{J^{LA}\left(i,j\right),J_{\theta }^{U}\left(i,j\right)\right\}$$

where $\left(i,j\right)$ is the pixel coordinate, θ represents three channels of the YCbCr color space, and $C_{\theta }$ aims to eliminate the superposition effect.

Studies have found that the color masking effect behaves actively for masking of luminance targets [53]. In other words, the masking effect increases with the increase in color uncertainty. Accordingly, the increase in the visibility threshold in the luminance component is more or less caused by the presence of the chrominance component in the color image. Therefore, the color uncertainty masking estimation $J^{U}$ can be modeled as

(17) $$J^{U}=\psi \cdot g\left(U\right)$$

where ψ is the masking effect estimation based on luminance predicted residuals [14] and $g\left(U\right)$ represents the gain control [54] of color uncertainty *U*, $g\left(U\right)=\tau_{1}\cdot \frac{U^{\tau_{2}}}{U^{2}+\tau_{3}}$, where $\tau_{1}$ is a proportionality constant; $\tau_{2}$ performs as an exponential parameter; and $\tau_{3}$ is a very small number, used to avoid a denominator of zero.

Since the visual energy of human eyes is concentrated in the area around the gaze point, the masking effect here is suppressed, while the masking effect in the unattended area is enhanced. According to analysis in introduction, the visual saliency is regarded as the adjustment factor for the masking estimation $J_{}^{M}$ to obtain the more humanized JND threshold $J_{}^{C}$.

(18) $$J_{}^{C}=J_{}^{M}\cdot W_{}^{S}$$

where $W_{}^{S}$ denotes the intensity of color saliency adjustment. As the region with saliency tolerates smaller distortion, the corresponding JND threshold is relatively lower, while the area with low perceived significance is not easy to notice by the human eye and its JND threshold is correspondingly larger. Based on this, color saliency adjustment intensity $W_{}^{S}$ is set.

(19) $$W_{}^{S}=\kappa_{1}-\exp\left(I-\kappa_{2}\right)$$

Among it, color saliency *I* has been normalized, and $\kappa_{1}$ is taken as 2 and $\kappa_{2}$ as 0.5 by subjective experiments. Therefore, adjusted by color saliency, the JND threshold is closer to real perception.

Finally, considering the different sensitivity of the human eye to different colors, perceptual weights are assigned to obtain the final JND estimation.

(20) $$J_{\theta }^{}=J_{\theta }^{C}\cdot W_{\theta }^{C}$$

where $W_{\theta }^{C}$ denotes the color sensitivity weight of the YCbCr three-channel [55].

## 4. Experimental Results and Analysis

To evaluate the superiority of the performance of the proposed JND model, objective and subjective quality evaluation experiments are implemented in this section and compared with the comparison models.

### 4.1. Noise Injection Method

The JND model is built to approach the actual HVS threshold and to avoid its over- or underestimation. When the difference in perceived quality between the original image and its corresponding JND-contaminated image is more indistinguishable, it means that the JND model performs better. Usually, JND models are used to add noise to the image, and a more accurate JND calculation model tends to hide more noise in a specific region, i.e., the corresponding JND value should be as large as possible, given the same subjective quality of the image. Concretely, JND-guided noise is added to images by

(21) $$F_{\theta }^{{}^{\prime }}\left(i,j\right)=F_{\theta }\left(i,j\right)+rand\left(i,j\right)\cdot J_{\theta }\left(i,j\right)$$

where *F* is the original color image, θ represents the three channels of YCbCr, and $F^{{}^{\prime }}$ is the noise-contaminated image. rand represents the bipolar random noise that is randomly decided to avoid the occurrence of noise change in the fixed pattern.

### 4.2. Ablation Experiments

**Figure 5:**
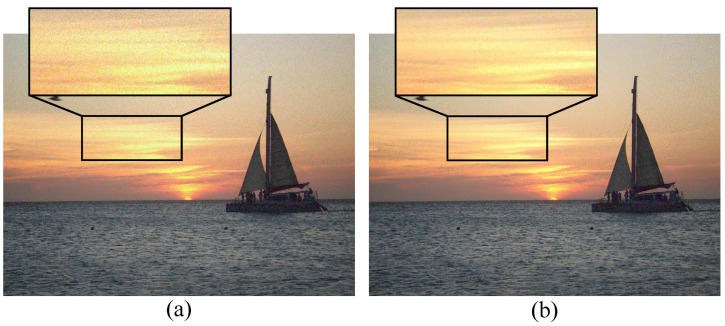
(**a**,**b**) The masking effect without color saliency adjustment and the masking effect with color saliency adjustment, respectively. Equal noise is added to both figures (PSNR is 26 dB for both).

To test the effectiveness of the proposed color saliency modulation and color sensitivity weights, a variable-controlled approach is selected here for the experiments. Figure 5 shows the comparison of the effect of uncertainty masking without and with color saliency modulation, and it can be clearly seen that the original uncertainty masking occurs in regions with high color complexity or irregular texture, such as the region in the seawater part. However, the region in the black box is close to the central part of the image and contains colors brighter in perception and significantly different from surroundings; so, this region attracts the human eye, i.e., the perceptual noise here can be easily detected. This is the reason for the poor perceptual quality of Figure 5a. After considering the color saliency adjustment masking effect, less noise is added, which results in a significant enhancement in perception in Figure 5b. This experiment proves that performance can be improved considering the visual saliency adjustment given equal amounts of noise.

**Figure 6:**
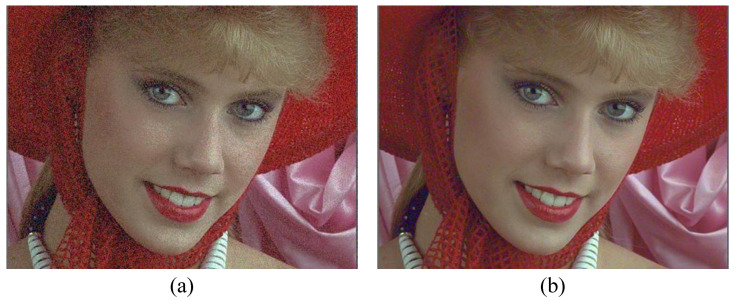
(**a**,**b**) The JND generation map without considering the color-sensitivity weighting and with considering the color-sensitivity weighting, respectively. Both maps add an equal amount of noise (PSNR is 26 dB for both).

Figure 6 compares the visual effect of adding JND directly to the original image, and adding color sensitivity to the three-channel weighted JND threshold in YCbCr color space. It is obvious to see that with the same amount of noise added, Figure 6a can feel obvious distortion, while Figure 6b is visually clear in the overall picture with almost no perceptible distortion. This is because the human eye is more sensitive to the luminance component than the color component, where a slight noise in the luminance component can be detected and the color component can accommodate more perceptual noise. If HVS assigns different perceptual weights to the three channels of YCbCr color space by adding less noise to the Y component and more noise to the Cb and Cr components, considering the different sensitivity of human eyes to color, the perceptual quality does not change significantly compared with the original images. This experiment proves that it is necessary to take color sensitivity into account for color image JND modeling.

### 4.3. Comparison Experiments

In this paper, ten images from each of the three color image databases are selected for testing TID2013 [56], IVC [57], and LableMe [58], with ’TID2013’ and ’IVC ’ being used for JND performance evaluation. The resolutions of both are 512 × 384 and 512 × 512, respectively. ’LableMe’ dataset is the selected high-resolution image dataset with a resolution of 1024 × 768. Here, the names are simplified to T1-T10, I1-I10, and L1-L10.

To measure the subjective quality of the images, this paper conducts a subjective quality assessment experiment with reference to the standard ITU-RBT.500-11. In each test, the original image and the noise-added image are presented side-by-side on the screen at the same time. Specifically, the original image was placed on the left as a reference standard, and the noise-injected image was played on the right screen in a random order for better comparison. The subjective scores were divided into four levels, indicating the degree of distortion compared with the original images, and the scoring criteria are shown in Table 2. Twelve subjects with good vision or vision correction were invited to rate the subjective quality in this experiment. The final MOS value is the average of the scores given by the twelve participants.

To obtain a reliable comparison, we choose the existing representative models for comparison, named as Wu2017 [15], Zeng2019 [59], Liu2020 [60], and Li2022 [61]. Wu2017 proposed pattern complexity, which divides the image into regular pattern regions and irregular pattern regions based on the diversity of pixel orientation in local regions, and integrates the pattern complexity and luminance contrast. Zeng2019 considered the masking effect of regular and irregular texture regions. The JND model of Liu2020 considered edge masking and image masking based on different image contents. Notably, both Zeng2019 and Liu2020 used a saliency factor for adjustment. Li2022 considered that the human eye is more sensitive to sharp edges than non-sharp edges, and proposed a screen-content spatial masking effect. These four models were chosen for comparison because they all considered the properties of each perceptual factor in detail, which is very similar in methodology to our proposed model that considers color image perceptual factors elaborately. Furthermore, these models are performed directly in the pixel domain, which makes the comparison more convincing.

Table 3 shows the comparison results of each model with objective metric PSNR and subjective metric MOS. The average PSNR metric indicates that the proposed JND model scores lower than Wu2017, Zeng2019, Liu2020, and Li2022 on all three datasets. It is found that for the TID2013 dataset, the JND models proposed by Wu2017, Zeng2019, and Liu2020 have higher PSNR, while the overall subjective quality score of the images is lower. The overall subjective quality of Li2022 is close to the proposed model, while the PSNR obtains 0.84 dB higher, indicating that its calculated JND threshold may be underestimated. The proposed model in this paper has the lowest PNSR and MOS score.

For the IVC dataset, Wu2017 image perception quality is poor, and subjective experiments find that it has significant distortion in some edge regions, especially for the face region; Zeng2019 and Liu2020 have significant noise in the color perception flat region, and both have relatively high PSNR, reaching 35.68 dB and 36.08 dB, respectively. The subjective perceptions of Li2022 and the proposed model are better, but the PSNR of the proposed model is 2.67 dB lower, indicating that the proposed model in this paper has better subjective perceptions while tolerating more perceptual noise.

**Figure 7:**
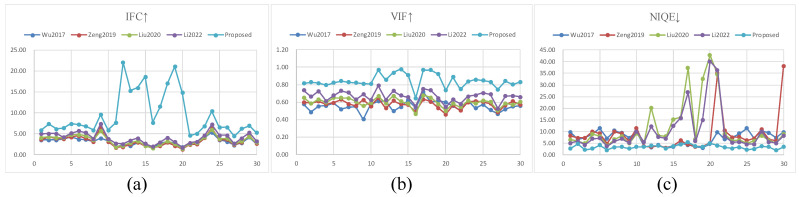
(**a**) The IFC score line graphs for each model in the 30-image sample; (**b**) the line graph of VIF score; (**c**) the line graph of NIQE score. ↑ represents that the bigger the value, the better the performance, and vice versa.

For the dataset LableMe, the PSNR values of the proposed JND model in this paper are significantly smaller than those of the other four comparison models, and the subjective quality is better. To evaluate the model performance more objectively, IFC [62], VIF [63], and NIQE [64] are also used in addition to the objective metric of PSNR. The results obtained from each model on the test dataset are compared with the original figure, and the comparison results are shown in Figure 7. It is clearly seen that the proposed model performs better compared with the comparison model. Collectively, it demonstrates that the subjective quality of the proposed JND model is in accordance with the real perceptual thresholds with better noise-masking ability.

**Figure 8:**
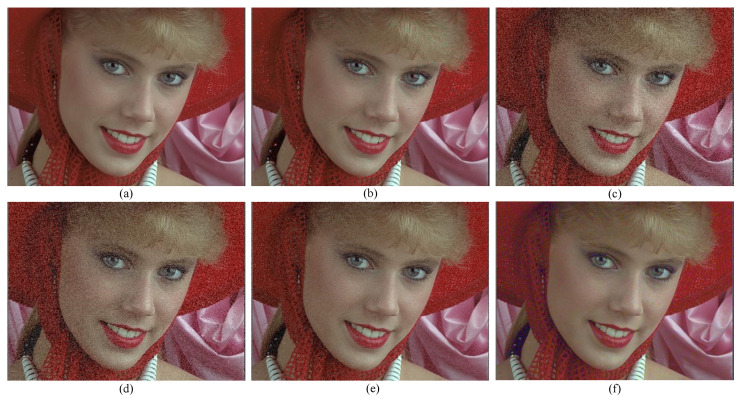
Schematic visual comparison of different JND models. (**a**) Original figure; (**b**) Wu2017; (**c**) Zeng2019; (**d**) Liu2020; (**e**) Li2022; (**f**) proposed JND model, all with a PSNR of 26 dB.

In addition, when viewing videos or pictures, people often pay extra attention to human subjects. Therefore, in order to directly and specifically compare the performance of different JND models, a classic color portrait image in a commonly used dataset is chosen here as an example. Figure 8a shows the original image, and Figure 8b–f show the noise-added images generated by Wu2017, Zeng2019, Liu2020, Li2022, and the proposed model in this paper, respectively. It can be seen from the results that the images generated by the models of Zeng2019, Liu2020, and Li2022 have perceptible noise in the flatter regions of the face, where the noise of the Zeng2019 model is very obvious and the noise of the Li2022 model generates images with less noise. Wu2017 has relatively less noise on the face, but there is obvious noise in the region around edges. The proposed JND model performs better, with visually sensitive face regions perceptually almost identical to the original image. The visual comparison results show that the proposed model is more consistent with the visual properties of the human eye in terms of overall perception.

We also compared with the latest pixel-domain color image JND model [31], which has not been open-sourced, on a unified dataset and the performance metrics published in the paper, with results in Table 4. From the results, it can be seen that our model Quality Score performs better. In summary, the proposed model performs better than the state-of-the-art color JND model.

## 5. Conclusions

In this paper, a pixel-domain JND model with elaborate color feature perception is proposed. Color-oriented features are firstly extracted. Based on this, color contrast intensity is proposed by analyzing interaction within color stimuli. Then, according to human visual perception to these features, we propose perceptually the color uncertainty and color saliency model by fusing related color features with information theory. Finally, to improve the conventional JND model, color saliency and uncertainty models are applied by serving as a masking and attention effect. Subjective and objective experiments validate the effectiveness of the proposed model, confirming that it has better perception performance with superior noise concealment capacity compared with reference works.

Nevertheless, the proposed model only concerns limited color feature, attaching the problem of color degradation during color region extraction. To achieve the accurate color-image JND, there is still a long way to go.

## Figures and Tables

**Table 1:** Existing color feature parameters and their corresponding perceptual effects.

Color Feature Parameter	Symbol	Effect
Color Complexity	$m_{c}$	Masking
Color Edge Intensity	$e_{c}$	Saliency
Color Distribution Position	$l_{c}$	Saliency
Color Perception Difference	$\chi_{c}$	Saliency
Color Distribution Dispersion	$v_{c}$	Masking
Color Area Proportion	$\rho_{c}$	Saliency

**Table 2:** Subjective quality scoring criteria.

Subjective Score	Scoring Criteria
0	The right figure has the same subjective quality as the left figure.
−1	The right image is slightly worse than the left image.
−2	The right image is of poorer subjective quality than the left image.
−3	The right image is much worse than the left image.

**Table 3:** Comparison of subjective and objective experimental results for image datasets.

Image Name	Wu2017 [15]		Zeng2019 [59]		Liu2020 [60]		Li2022 [61]		Proposed	
	PSNR (dB)	MOS	PSNR	MOS	PSNR	MOS	PSNR	MOS	PSNR	MOS
T1	36.51	−0.32	35.85	−0.30	36.29	−0.30	31.00	−0.20	32.03	−0.06
T2	35.47	−0.30	35.27	−0.30	36.34	−0.26	31.72	−0.16	32.23	−0.06
T3	36.39	−0.24	35.78	−0.22	36.32	−0.24	32.04	−0.18	28.94	−0.10
T4	31.51	−0.22	34.74	−0.12	33.55	−0.08	32.25	−0.08	31.58	−0.06
T5	35.21	−0.24	36.25	−0.26	36.75	−0.18	34.67	−0.10	31.92	−0.10
T6	33.99	−0.32	36.43	−0.28	35.35	−0.22	34.92	−0.10	34.34	−0.08
T7	33.80	−0.34	33.41	−0.40	33.80	−0.36	29.35	−0.34	28.25	−0.14
T8	34.69	−0.28	36.84	−0.24	37.32	−0.18	34.80	−0.12	34.06	−0.06
T9	34.06	−0.16	36.38	−0.22	35.14	−0.18	33.36	−0.10	29.81	−0.08
T10	36.95	−0.24	36.52	−0.30	37.21	−0.26	32.52	−0.16	35.01	−0.06
Avg	34.86	−0.27	35.75	−0.26	35.81	−0.23	32.66	−0.15	**31.82**	**−0.08**
I1	35.90	−0.28	36.16	−0.22	37.97	−0.16	37.09	−0.14	30.81	−0.12
I2	31.40	−0.40	35.62	−0.30	35.44	−0.30	32.46	−0.20	31.71	−0.08
I3	34.57	−0.22	36.52	−0.22	37.07	−0.18	35.66	−0.12	30.48	−0.10
I4	33.92	−0.34	34.21	−0.24	34.70	−0.20	29.58	−0.12	27.55	−0.08
I5	34.76	−0.22	34.52	−0.20	35.52	−0.16	29.81	−0.12	27.76	−0.06
I6	33.17	−0.26	36.39	−0.22	36.44	−0.12	34.77	−0.10	31.13	−0.10
I7	34.87	−0.40	35.67	−0.30	37.21	−0.22	33.57	−0.10	30.94	−0.06
I8	35.90	−0.34	36.01	−0.30	37.53	−0.18	33.72	−0.08	33.24	−0.12
I9	28.62	−0.14	36.49	−0.06	33.26	−0.06	33.21	−0.06	29.60	−0.06
I10	36.16	−0.24	35.21	−0.14	35.69	−0.10	30.69	−0.08	30.66	−0.08
Avg	33.93	−0.28	35.68	−0.22	36.08	−0.17	33.06	−0.11	**30.39**	**−0.09**
L1	38.34	−0.20	38.30	−0.14	38.04	−0.12	37.58	−0.04	32.00	−0.06
L2	36.72	−0.18	33.33	−0.18	33.58	−0.14	29.13	−0.08	27.87	−0.08
L3	34.09	−0.18	36.93	−0.16	37.89	−0.14	35.14	−0.08	30.96	−0.06
L4	34.83	−0.24	35.62	−0.18	35.84	−0.12	33.07	−0.10	34.01	−0.14
L5	37.52	−0.24	35.35	−0.24	35.41	−0.22	30.80	−0.12	30.17	−0.10
L6	34.71	−0.16	31.87	−0.14	31.56	−0.10	25.96	−0.08	24.49	−0.06
L7	40.20	−0.16	37.66	−0.14	38.30	−0.08	35.43	−0.08	35.82	−0.08
L8	36.47	−0.28	36.70	−0.30	37.08	−0.24	34.30	−0.10	33.83	−0.08
L9	37.62	−0.26	35.02	−0.26	35.51	−0.22	31.69	−0.16	31.31	−0.10
L10	37.45	−0.18	33.02	−0.32	33.22	−0.28	28.10	−0.20	28.21	−0.12
Avg	36.80	−0.21	35.38	−0.21	35.64	−0.17	32.12	−0.10	**30.87**	**−0.09**

Bolded data characterizes optimal performance.

**Table 4:** Comparison performance between Jin2022 and the Proposed in Database CSIQ.

	Index	MAD [65]	VIF [63]	VSI [66]
Model				
**Jin2022 [31]**		30.8754	0.5918	0.9958
**Proposed**		15.7638	0.8089	0.9973
**Reference**		0	1	1

## Data Availability

Not applicable.
